# Utilisation of Design of Experiments Approach to Optimise Supercritical Fluid Extraction of Medicinal Cannabis

**DOI:** 10.1038/s41598-020-66119-1

**Published:** 2020-06-04

**Authors:** Simone Rochfort, Ashley Isbel, Vilnis Ezernieks, Aaron Elkins, Delphine Vincent, Myrna A. Deseo, German C. Spangenberg

**Affiliations:** 1 Agriculture Victoria, AgriBio, 5 Ring Road, Bundoora, Victoria, 3083 Australia; 2 PharmOut, Burwood East, Victoria, 3151 Australia; 3 Present Address: La Trobe Institute for Agriculture and Food, School of Life Sciences, Department of Animal, Plant and Soil Sciences, La Trobe University, 5 Ring Road, Bundoora, Victoria, 3083 Australia

**Keywords:** Secondary metabolism, Chemistry

## Abstract

Carbon dioxide supercritical fluid extraction (CO_2_ SFE) is a clean and cost-effective method of extracting cannabinoids from cannabis. Using design of experiment methodologies an optimised protocol for extraction of medicinal cannabis bud material (population of mixed plants, combined THC:CBD approximately 1:1.5) was developed at a scale of one kg per extraction. Key variables investigated were CO_2_ flow rate, extraction time and extraction pressure. A total of 15 batches were analysed for process development using a two-level, full factorial design of experiments for three variable factors over eleven batches. The initial eleven batches demonstrated that CO_2_ flow rate has the most influence on the overall yield and recovery of the key cannabinoids, particularly CBD. The additional four batches were conducted as replicated runs at high flow rates to determine reproducibility. The highest extraction weight of 71 g (7.1%) was obtained under high flow rate (150 g/min), with long extraction time (600 min) at high pressure (320 bar). This method also gave the best recoveries of THC and CBD. This is the first study to report the repeated extraction of large amounts of cannabis (total 15 kg) to optimise the CO_2_ SFE extraction process for a pharmaceutical product.

## Introduction

Cannabis is an herbaceous flowering plant of the *Cannabis* genus (Rosale) that has been used for its fibre and medicinal properties for thousands of years^1,2^. In recent decades medicinal cannabis has become legal in several jurisdictions and the possibility of legalisation is being explored in many more^3–8^. The chemistry of cannabis is rich and varied; it includes phytocannabinoids, terpenes and phenolics, and each of these classes contain compounds with biological activity^9^. The cannabinoids, in particular, Δ^9^-tetrahydrocannabinol (THC) and cannabidiol (CBD) have been the main focus of bioactive research. These cannabinoids are naturally present *in planta* as their acid analogues Δ^9^-tetrahydrocannabinolic acid (THCA) and cannabidiolic acid (CBDA). THCA and CBDA are the major cannabinoids found in cannabis although more than 90 cannabinoids have been reported^9^. The acids degrade naturally to the corresponding neutral species at a slow rate via non-enzymatic processes^10^. Decarboxylation is facilitated by heating (e.g. when smoked) (Citti *et al*. 2018). Many medicinal cannabis preparations are consumed in an oil or are made from an extracted resin and not smoked. In this case, the dried cannabis material must be ‘cured’ prior to, or during, extraction (*i.e*. heated for a specific time and temperature to ensure maximum decarboxylation). THCA is the main psychoactive cannabinoid followed by cannabigerolic acid (CBGA).

Medicinal cannabis is consumed by patients using a variety of methods including smoking, vaping and consuming infused oils or other edible products. One of the issues associated with smoking or vaping is that the dose the patient receives is not reliable due to both the variability in plant product and the way patients inhale and hold vapour^11^. Vaping and smoking also have well known negative health effects and is particularly undesirable for juvenile patients. For this reason, the production of an extract that can be formulated in various ways is becoming more popular. The cannabinoids are non-polar compounds with low water solubility and can be extracted using a range of different organic solvents including hydrocarbons (e.g. hexane) and alcohols (e.g. ethanol). Extractions using these solvents can be efficient but depending on the final product, can impact regulation and require additional testing. For instance, residual solvent must be defined for medicines under good manufacturing practice (GMP). These solvents can also be costly and due to their toxicity, environmental risk and flammability are less desirable for large scale extractions.

Supercritical fluid extraction (SFE) can eliminate the need for these organic solvents during the manufacture of medicinal cannabis extracts. Carbon dioxide (CO_2_) is the main solvent in SFE. It has GRAS (generally recognised as safe) status and evaporates from the extract when exposed to normal atmospheric conditions. The majority of the published studies on SFE of cannabis are either at small/analytical scale^12^ or on hemp material that contains low levels of cannabinoids and very little THC^13–18^. There have been studies investigating the solubility of THC and CBN (cannabinol, a degradation product of THC) in supercritical CO_2_ suggesting that temperature and pressure are critical parameters^19,20^. A more recent study evaluated the use of the Waters Bio-botanical extraction system with the use of ethanol as a co-solvent with CO_2_ extraction using four cannabis samples (500 g each) with THCA:CBDA content of 15:1 or more. The authors noted extraction efficiency was highly linked to pressure as well as cannabinoid content of the plant material^21^.

This study was undertaken as part of our efforts to obtain a GMP license to produce pharmaceutical grade medicinal cannabis. In order to simplify the regulatory process, and produce a cleaner product, we chose to avoid the use of co-solvents such as ethanol. Process development (PD) is part of GMP. The aim of PD to ensure that the factors underlying any manufacturing protocols are well understood so that any potential source of product variation can be better managed to ensure a safe and consistent pharmaceutical product. As part of GMP PD we used design of experiments (DOE) methodology to develop an extraction method for medicinal cannabis. The aim of DOE was to explore the factors influencing the extraction of cannabinoids and optimise the process for extraction of cannabis material at a pharmaceutical scale.

DOE is commonly used to optimise engineering processes and to determine which process inputs have a significant effect on process outputs. In a factorial design each variable, or factor, is investigated at predefined levels. With a two-level factorial design each factor can be one of two values. For continuous variables, this is a high and a low level of the variable under investigation^22^. Importantly, the methodology can also determine if there are interactions between the factors in the process. This allows for more rapid optimisation of processes than if single variables were considered independently. This methodology has been used to investigate factors important in extraction of biomaterials. For example, Prasad *et al*. explored the effect of five factors (pH, ethanol concentration, temperature, time and liquid to solid ratio) on four responses (extract yield, antioxidant capacity, phenolics and flavonoids) for the extraction of the Malaysian fruit, *Mangifera pajang*^23^. Lee *et al*. analysed similar factors to optimise the recovery of phenolics, flavonoids and anti-oxidants from palm-kernel by-products^24^. A review of the literature by Reverchon and De Marco demonstrated that CO_2_ flow rate, extraction pressure and extraction time are the critical parameters in SFE for the extraction of natural materials^25^. Therefore, these were the factors considered in this study.

## Results and Discussion

The factors considered in this study were the CO_2_ flowrate, the extraction time, extraction pressure, recoveries of CBD and THC and the final extract weight (Table 1). The range of values for factors was based on review of the literature and the recommendations of the instrument manufacture (Waters). For example, if the flow rate is too low the system will not function properly and if it is too high it will shut down due to over pressurisation. The temperature of the extraction vessels was set at 60 °C based on the recommendation of the manufacture. If we consider %CBD recovery (response 1 in Table 1), from the initial two-level factorial extractions (runs 1 to 8), the four lowest recovery rates correspond to the four low flow rate runs; i.e., runs 2, 3, 4 and 6. Only when combined with maximum time and pressure (run 6), did low flow rates produce CBD recoveries which approached the high recoveries produced at high flow rate.

**Table 1:** Experimental design and results of extraction (rows coloured by experiment: white indicates the initial factorial designed runs, light grey indicates mid-point runs, dark grey indicates repeat runs).

Run	Factor A	Factor B	Factor C	Response 1	Response 2	Response 3
	CO_2_ Flowrate (g/min)	Extraction time (min)	Extraction pressure (bar)	CBD % Recovery	THC % Recovery	Extract weight (g)
1	150	600	320	101.1	98.6	71.0
2	40	600	150	41.9	16.7	27.5
3	40	240	320	7.5	5.0	4.2
4	40	240	150	22.0	9.0	9.1
5	150	240	320	95.9	66.8	55.1
6	40	600	320	78.1	84.0	55.9
7	150	600	150	107.1	73.6	56.3
8	150	240	150	90.2	75.6	50.8
9	95	420	235	82.7	98.2	62.7
10	95	420	235	68.3	82.9	57.8
11	95	420	235	66.7	82.6	57.2
12	150	600	320	89.2	97.6	68.1
13	150	240	320	81.4	93.0	62.7
14	150	600	150	77.2	93.5	58.3
15	150	240	150	79.1	74.1	47.7

**Figure 1:**
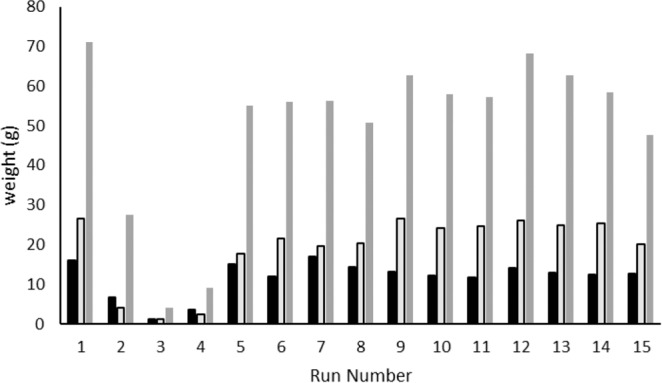
Comparison of CBD, THC and total resin yield for each PD run. CBD: solid black bars, THC: light grey bars outlined in black, Total Extract Weight: solid grey bars.

Generally speaking, of the initial two-level factorial runs (1 to 8), the high flow rates (150 g/min) produced excellent CBD recovery, with all exceeding 90% of theoretical yield. The yields of each run are compared in Fig. 1.

When analysed statistically, the CO_2_ flow rate is shown to have largest effect on CBD recovery. Extraction pressure had the least overall impact to CBD recovery even when combined with other factors (Table 2).

**Table 2:** Result of statistical analysis of Response 1– %CBD Recovery.

Term	Standardised Effect	% Contribution
A: CO_2_ Flowrate	52.77	62.86
B: Extraction time	26.12	15.40
C: Extraction pressure	6.31	0.90
AB	−19.12	8.26
AC	−3.67	0.30
BC	12.43	1.67
ABC	−12.93	1.96

The effect of factors on THC recovery is not as clear cut as for CBD. While three of the four lowest recoveries were obtained in low flow rate runs, the 2nd best result was also recorded on a flow rate run at 95 g/min. The other factors (time and pressure), and in particular, interactions between these factors also appear to have an impact (Table 3)

**Table 3:** Result of statistical analysis of Response 2– %THC Recovery.

Term	Standardised Effect	% Contribution
A: CO_2_ Flowrate	55.42	52.64
B: Extraction time	24.84	10.57
C: Extraction pressure	18.12	5.63
AB	−14.95	3.83
AC	−10.92	2.05
BC	20.20	4.37
ABC	−15.45	1.46

When analysed statistically, the CO_2_ flowrate has the highest effect, but there are a number of other significant effects (Table 3).

Extraction weight seems to be most significantly influenced by CO_2_ flow rate and extraction time, with smaller influence from extraction pressure. Statistical analysis (Table 4) confirms this.

**Table 4:** Result of statistical analysis of Response 3– % Extraction weight.

Term	Standardised Effect	% Contribution
A: CO_2_ Flowrate	34.57	50.54
B: Extraction time	22.20	20.84
C: Extraction pressure	11.90	5.98
AB	−12.85	6.98
AC	−0.40	0.01
BC	8.98	1.96
ABC	−7.68	1.04

The highest extraction weight of 71 g (7.1%) was obtained under high flow rate (150 g/min), with long extraction time (600 min) at high pressure (320 bar). This method also gave the best recoveries of THC and CBD (Table 3). The lowest extraction weight of 4.2 g (0.42%) was obtained with low flow rate (40 g/min) and short extraction time (240 min) even though the extraction pressure was high (320 bar). Runs 5–8 produced very similar quantities (50.8 g to 56.3 g) even though the extraction times were 240 min and 600 min, indicating that a longer extraction time did not necessarily increase the amount of extract. The three centre point runs (9–11) produced extraction weights as good or better than runs 2–8. The last four runs (12–15) were duplicates of runs 1, 5, 7 and 8. With the exception of run 13, the yields from the duplicate runs were similar (within 3 g).

When the concentration of CBD and THC in each extract are compared, it is clear that the different run conditions do affect CBD and THC differently (Table 5). The ratio of CBD:THC varies from 0.5 to 1.6. The CBD:THC ratio in the cured material is approximately 0.7 and the data shows that this ratio can be increased if the flow rate is decreased, however, there is significant cost to yield (a maximum yield of 27.5 g compared to 71.0 g)

**Table 5:** Quantity of CBD and THC in the extract and the ratio between them in each run.

Run	CBD in extract(mg/g)	THC in extract(mg/g)	CBD:THC	total CBD(g)	total THC(g)	% THC and CBD in extract	Extract weight (g)
1	113.5	187.6	0.6	16.1	26.6	60.1	71.0
2	120.8	761.1	1.6	6.6	4.2	39.3	27.5
3	133.2	149.5	0.9	1.1	1.3	57.1	4.2
4	191.7	132.3	1.4	3.5	2.4	64.8	9.1
5	137.8	161.9	0.9	15.2	17.8	59.9	55.1
6	107.6	193.5	0.6	12.0	21.6	60.1	55.9
7	150.7	174.8	0.9	17.0	19.7	65.2	56.3
8	141.6	200.2	0.7	14.4	20.3	68.3	50.8
9	105.5	211.5	0.5	13.2	26.5	63.3	62.7
10	105.1	208.5	0.5	12.2	24.1	62.8	57.8
11	103.5	215.8	0.5	11.8	24.7	63.8	57.2
12	103.6	191.3	0.5	14.1	26.1	59.0	68.1
13	103.2	199.0	0.5	12.9	25.0	60.4	62.7
14	106.7	218.2	0.5	12.4	25.4	64.8	58.3
15	132.8	210.0	0.6	12.7	20.0	68.6	47.7

Despite this reduction in extraction weight, there may be value in this approach if there is a need for medicinal purposes. For example, high CBD and low (or no) THC is currently desirable for the treatment of epilepsy in children^26^. For a plant biomass exhibiting high CBD and low THC, this method may be optimised further so that low levels of THC could be removed. Based on the current study the optimised method for maximising the levels of either CBD or THC in the extract would be different. Analysis using Design Expert software suggests that extraction pressure would have the greatest effect in this case (Table 6).

**Table 6:** Optimisation of parameters to maximise CBD or THC.

Target	CO_2_ Flowrate (g/min)	Extraction time (min)	Extraction pressure (bar)	Confidence %
High THC, high extraction weight with minimal CBD	150	400	320	82.1
High CBD, high extraction weight with minimal THC	150	420	150	75.5
Maximised Yield (all cannabinoids)	150	600	320	99.5

These findings are in line with the literature on cannabinoid solubility in supercritical CO_2_. Perrotin-Brunel *et al*. found that CBD is more soluble than THC at both 42 °C and 52 °C over pressures from 110 bar to 210 bar^19,20,27^. They also note that CBD and THC might be separated by using a two-step process, first extracting CBD at 130 bar then extracting THC at 320 bar (close to the parameters suggested in Table 6)^27^.

Of course, cannabinoids are not the only metabolites extracted from cannabis biomass. Attard *et al*. demonstrated that in the SFE of hemp waste fatty acids, fatty aldehydes, wax esters, alkanes, sterols and n-policosanols were extracted along with CBD and that different parameters could alter the amount of these other metabolites^17^. In our study between 32% and 61% of the extract is made up of other components. While our study did not investigate these other metabolites, it is clear that our extraction variables also affect the amounts of these metabolites in our final extract. Different cannabis plants will have different levels of the cannabinoids as well as these other metabolites. It is therefore anticipated that for GMP extraction of any new biomaterial PD would need to be repeated and extraction optimised for that specific cannabis material.

## Conclusion

CBD and THC can be extracted with high efficiency from cured cannabis biomass using supercritical CO_2_ without a co-solvent. The design of experiments approach allowed efficient sampling of CO_2_ flow rates, CO_2_ pressures and extraction times to assess the impact these variables have on recovery of CBD, recovery of THC and total extract yield. The extraction of CBD is largely dependent on flow rate while, the extraction of THC is dependent on both flow rate and extraction time. In terms of cannabinoid recoveries and extract yield the optimal extraction parameters for the biomass used in this study was a flow rate of 150 g/min, an extraction time of 600 min and an extraction pressure of 320 bar.

## Materials and methods

### Chemicals and equipment

The CO_2_ was food grade (99.5% with less than 17.5 ppm water) and obtained from CoreGas (Thomastown, Victoria, Australia). All HPLC grade reagents, water with 0.1% formic acid (mobile phase A), acetonitrile with 0.1% formic acid (mobile phase B) and methanol were obtained from Fisher Scientific (Fair Lawn, New Jersey, USA). Primary standards for CBDA and THCA in acetonitrile, and CBD, CBN, CBC, THC in methanol, at 1000 μg/mL, were purchased from Novachem Pty Ltd (Heidelberg West, Victoria, Australia) as distributor for Cerilliant Corporation (Round Rock, Texas, USA). Glass marbles (12 mm, clear, MoreWine, CA) were used to top up the baskets to fill the void space after packing the cannabis material.

### Cannabis material

Dried and ground plant material was obtained from the Victorian Government Medicinal Cannabis Cultivation Facility. Biomass was combined from cannabis plants of varying genotype and chemotype to provide 18 kg of material of composite material with CBD:THC ratio of approximately 1:1.5. Coarsely ground biomass samples were cured at 120 °C for 2 hours facilitating the conversion of CBDA to CBD and THCA to THC. Subsamples of the cured material were ground to a fine powder and analysed to provide baseline cannabinoid content data using UHPLC analytical methods as described previously^28^.

### Supercritical carbon dioxide extraction of cannabis biomass

**Figure 2:**
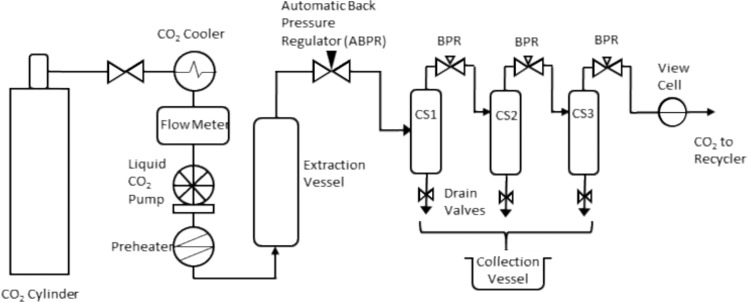
Schematic of the SFE set up. CS = cyclonic separator (cyclone), BPR = manually adjusted back pressure regulator (adapted from Rovetto and Aieta^21^).

The Waters Bio-Botanical extraction system^29^ was used throughout PD (Schematic, Fig. 2). The system is commercially available and was fitted with two extraction vessels (5 L, though only one was used for PD), three collection vessels (cyclones) and a recycler to recover CO_2_. The extraction vessels were equipped with removable baskets that facilitate the transfer of cannabis to and from the system. The extraction vessels were maintained at 60 °C (via heated jacket) for all runs. The extraction pressure is maintained automatically by the ABPR (automatically actuated back pressure regulator) which is an actuated needle valve. The three collection vessels are independently heated and fitted with manual back pressure regulators. These regulators were set as suggested by the manufacturer to 750 psi, 1200 psi and 2000 psi and the temperatures set to 35 °C, 50 °C and 55 °C, respectively. The system is controlled by ChromScope v1.6 (Waters).

We were not interested in assessing the capability of the system to separate different fractions and so the extract in each cyclone was combined prior to analysis. For each PD run the baskets were packed with the cured cannabis biomass (1.0 kg). The material was manually packed tight into the baskets using a custom-made stainless-steel plunger. The remaining space in the basket was filled with food grade glass marbles. The extraction programs were set up as described in Table 4.

Cannabinoid content of the resin was determined using UHPLC analytical methods described previously^28^. The %CBD and %THC recoveries were calculated by comparing the concentration in the resin and the total cannabinoid extracted to the amount available in the biomass.

### Experimental design

PD was designed and the data interpreted using Design Expert (Stat-Ease, version 10), which is a commercial, off-the-shelf Design of Experiments package. The system uses standard statistical methodology, such as analysis of variance (ANOVA), to determine the factors influencing responses that are critical to the final product (e.g. extraction weight). Additionally, it considers interactions between the different factors to determine if there are any synergistic effects between them.

The effects of interactions are represented by factor identifications (i.e. AB is the interaction effect of factors A and B). The standardised effect represents the degree to which a factor exhibits a positive or negative effect on the response (as indicated by the sign of the number), while the % contribution estimates the factor’s overall influence on the response. The software was used to model various combinations of factors to determine process optimisation. Literature demonstrates that CO_2_ flow rate, extraction pressure and extraction time are the critical parameters in SFE^25^.

Therefore, the three factors chosen for variance were:

- Factor A: CO_2_ flow rate, 40–150 g/min
- Factor B: Extraction pressure, 150–320 bar
- Factor C: Extraction time, 4–10 hours

The parameters used to assess extraction efficiency were:

- Response 1: %CBD recovery
- Response 2: %THC recovery
- Response 3: Extraction weight (g)

A total of 15 extractions were analysed for PD using a two-level, full factorial design of experiments for three variable factors over eight runs (1 to 8, Table 1), three mid-point runs (9 to 11, Table 1) and four additional extractions were conducted as replicated runs at high flow rates to determine reproducibility (runs 12 to 15, Table 1).

## References

[1] Brunner TF (1973). Marijuana in ancient greece and rome? The literary evidence. Bull His Med.

[2] ElSohly MA, Slade D (2005). Chemical constituents of marijuana: The complex mixture of natural cannabinoids. Life Sci..

[3] Ghiabi M, Maarefvand M, Bahari H, Alavi Z (2018). Islam and cannabis: Legalisation and religious debate in Iran. Int. J. Drug Policy.

[4] Cruz JM, Fernanda Boidi M, Queirolo R (2018). The status of support for cannabis regulation in Uruguay 4 years after reform: Evidence from public opinion surveys. Drug Alcohol Rev..

[5] Bone M, Potter G, Klein A (2018). Introduction: cultivation, medication, activism and cannabis policy. Drugs and Alcohol Today.

[6] Bradford AC, Bradford WD (2017). Factors driving the diffusion of medical marijuana legalisation in the United States. Drugs-Education Prevention and Policy.

[7] Wilkins C (2016). After the legalisation of cannabis: the Cannabis Incorporated Society (CIS) regulatory model for recreational cannabis in New Zealand. N.Z. Med. J..

[8] Wright, R. Cannabis Industry Report. 40 (EverBlu Capital, https://www.everblucapital.com/wp-content/uploads/2017/11/EverBlu-Research-Cannabis-Industry-Report.pdf, 2017).

[9] Andre, C. M., Hausman, J.-F. & Guerriero, G. Cannabis sativa: The Plant of the Thousand and One Molecules. *Front Plant Sci* **7**, 10.3389/fpls.2016.00019 (2016). 10.3389/fpls.2016.00019 PMC4740396 26870049

[10] Flores-Sanchez IJ, Verpoorte R (2008). Secondary metabolism in Cannabis. Phytochem. Rev..

[11] Lewis MM, Yang Y, Wasilewski E, Clarke HA, Kotra LP (2017). Chemical Profiling of Medical Cannabis Extracts. ACS Omega.

[12] Omar J, Olivares M, Alzaga M, Etxebarria N (2013). Optimisation and characterisation of marihuana extracts obtained by supercritical fluid extraction and focused ultrasound extraction and retention time locking GC-MS. J. Sep. Sci..

[13] Da Porto C, Decorti D, Natolino A (2014). Separation of aroma compounds from industrial hemp inflorescences (Cannabis sativa L.) by supercritical CO2 extraction and on-line fractionation. Ind. Crops. Prod..

[14] Aladic K (2014). Cold Pressing and Supercritical CO2 Extraction of Hemp (Cannabis sativa) Seed Oil. Chem. Biochem. Eng. Q..

[15] Da Porto C, Natolino A, Decorti D (2015). Effect of ultrasound pre-treatment of hemp (Cannabis sativa L.) seed on supercritical CO2 extraction of oil. J Food Sci Tech Mysore.

[16] Brighenti V, Pellati F, Steinbach M, Maran D, Benvenuti S (2017). Development of a new extraction technique and HPLC method for the analysis of non-psychoactive cannabinoids in fibre-type Cannabis sativa L. (hemp). J. Pharm. Biomed. Anal..

[17] Attard TM (2018). Utilisation of supercritical fluids for the effective extraction of waxes and Cannabidiol (CBD) from hemp wastes. Ind. Crops. Prod..

[18] Devi V, Khanam S (2019). Comparative study of different extraction processes for hemp (Cannabis sativa) seed oil considering physical, chemical and industrial-scale economic aspects. J Clean Prod.

[19] Perrotin-Brunel H (2010). Solubility of Cannabinol in Supercritical Carbon Dioxide. J. Chem. Eng. Data.

[20] Perrotin-Brunel H (2010). Solubility of Delta(9)-tetrahydrocannabinol in supercritical carbon dioxide: Experiments and modeling. J. Supercrit. Fluids.

[21] Rovetto LJ, Aieta NV (2017). Supercritical carbon dioxide extraction of cannabinoids from Cannabis sativa L. J. Supercrit. Fluids.

[22] Carlson, R. in Data Handling in Science and Technology Vol. 8 (ed Rolf Carlson) 89-122 (Elsevier, 1992).

[23] Prasad KN, Kong KW, Ramanan RN, Azlan A, Ismail A (2012). Selection of Experimental Domain using Two-Level Factorial Design to Determine Extract Yield, Antioxidant Capacity, Phenolics, and Flavonoids from Mangifera pajang Kosterm. SS&T.

[24] Lee WX (2015). Two level half factorial design for the extraction of phenolics, flavonoids and antioxidants recovery from palm kernel by-product. Ind. Crops. Prod..

[25] Reverchon E, De Marco I (2006). Supercritical fluid extraction and fractionation of natural matter. J. Supercrit. Fluids.

[26] Tzadok M (2016). CBD-enriched medical cannabis for intractable pediatric epilepsy: The current Israeli experience. Seizure.

[27] Perrotin-Brunel H (2010). Solubility of non-psychoactive cannabinoids in supercritical carbon dioxide and comparison with psychoactive cannabinoids. J. Supercrit. Fluids.

[28] Elkins AC, Deseo MA, Rochfort S, Ezernieks V, Spangenberg G (2019). Development of a validated method for the qualitative and quantitative analysis of cannabinoids in plant biomass and medicinal cannabis resin extracts obtained by super-critical fluid extraction. J Chromatogr B Analyt Technol Biomed Life Sci.

[29] Waters, E. J. SFE Bio-Botanical Extraction System, https://www.waters.com/waters/en_US/For-SFE-extraction-and-CO2-extraction/nav.htm?cid=134826287&locale=en_US> (2019).

